# Adherence to prescribed antihypertensive medication among patients with depression in the United States

**DOI:** 10.1186/s12888-022-04424-x

**Published:** 2022-12-05

**Authors:** Quanjun Liu, Haochen Wang, Anbang Liu, Cheng Jiang, Weiya Li, Huan Ma, Qingshan Geng

**Affiliations:** 1 Department of Cardiology, Guangdong Cardiovascular Institute, Guangdong Provincial People’s Hospital, Guangdong Academy of Medical Sciences, 106 Zhongshan er Road, Guangzhou, Guangdong China; 2 School of Medicine, South China University of Technology, Guangzhou, 510006 Guangdong China; 3 Shenzhen People’s Hospital, No. 1017, Dongmen North Road, Shenzhen, 518000 Guangdong China

**Keywords:** Depression, Hypertension, Medication adherence

## Abstract

**Background:**

Hypertensive patients with depression have a higher mortality rate and a worse prognosis compared with hypertensive only. Depression may reduce medication adherence in hypertension patients.

**Methods:**

This study includes respondents in the National Health and Nutritional Examination Survey (NHANES) database from 2005 to 2018 who had previously been diagnosed with hypertension. Medication adherence was defined as taking medication as recommended by a physician. The depressive state was assessed using the patient health questionnaire (PHQ)-9.

**Results:**

Nine thousand one hundred eighty-six respondents were included in the analysis. Medication adherence was associated with depression (odds ratio [OR]: 1.48, 95% confidence interval [CI]: 1.26 to1.75) and depression score (OR: 1.04 per each point increase, 1.03 to 1.05) in the unadjusted analyses. After adjusting for clinical and socioeconomic/demographic factors, there were significant statistical correlations between depression score and medication adherence (aOR: 1.02 per each point increase, 1.00 to 1.03, *p* < 0.05), but there was no significant statistical correlation between depression and medication adherence (*p* > 0.05). It was still statistically significant relationships between sex, age, body mass index (BMI), race, marital status, and health insurance with medication adherence after adjusted socioeconomic/demographic factors.

**Conclusion:**

Depression was marginally associated with poor medication adherence in hypertensive patients, and the correlation increased with depression degree. Moreover, socioeconomic/demographic factors have an independent impact on medication adherence including sex, age, BMI, race, marital status, and health insurance.

**Supplementary Information:**

The online version contains supplementary material available at 10.1186/s12888-022-04424-x.

## Introduction

Hypertension is common in the United States (US) and is often poorly controlled [1]. Uncontrolled hypertension is a risk factor for many leading causes of death, including myocardial infarction and stroke [2]. Only around half of the patients with hypertension are treated [1]. Lack of treatment or poor adherence to prescribed antihypertensive medications is a significant contributor to the incidence of uncontrolled hypertension [3].

Many factors may influence medication adherence, including sociodemographic characteristics, disease education, and national policies that alter access to medication [4]. The influence of psychological factors on medication adherence may also play a role, particularly in depression [5–8]. Hypertension and depression are risk factors for cardiovascular morbidity and mortality, and people with both conditions have a worse prognosis than those with hypertension alone [9, 10]. Previous studies have shown the prevalence of depression among patients with hypertension to be between 26 and 57% [11–13]. Compared with nondepressed patients, the possibility was higher that depressed patients would be noncompliant with medical treatment recommendations [8].

Although the relationship between depression and medication adherence has been explored in recent years, depression is associated with poor medication adherence, and other factors related to medication adherence have been analyzed. However, studies on the relationship between depression and antihypertensive medication adherence have not been clarified well, and the results have been inconsistent [5, 14–18].

In hypertension patients, we hypothesize that depression negatively affects medication adherence; other sociodemographic factors can also influence medication adherence. The purpose of this study was to explore the relationship between depression, sociodemographic characteristics and medication adherence in National Health and Nutritional Examination Survey (NHANES) respondents (2005–2018) with hypertension and who were prescribed antihypertensive medications [19].

## Methods

### Sample

The NHANES is a biennial, cross-sectional, multistage, stratified, clustered probability survey of the civilian, non-institutionalized US population conducted by the National Center of Health Statistics. This analysis used data extracted from the 2005–2018 surveys for respondents (≥18 years old) who reported being told more than twice by a healthcare professional that they had high blood pressure and needed to take prescribed medicine because of high blood pressure. Standardized questions to this effect were included in the NHANES home questionnaire, implemented by trained interviewers using the Computer-Assisted Personal Interview (CAPI) system. All data were reviewed by the NHANES field office staff for accuracy and completeness. The NHANES protocol was approved by the National Center for Health Statistics Ethics Review Board, and written informed consent was obtained from all participants. In addition, this study was approved by the Ethical Review Board of Guangdong Provincial People’s Hospital. All NHANES data used in our analysis are publicly available at https://www.cdc.gov/nchs/nhanes/.

### Objective

The primary objective of this analysis was to look at the correlation between medication adherence and depression in hypertension patients. A secondary objective was to look at the influences of medication adherence by other sociodemographic factors.

### Depressive symptoms

An overall depression score was obtained from NHANES respondents using the Patient Health Questionnaire (PHQ)-9. The PHQ-9 is a self-administered version of the PRIME-MD diagnostic instrument for common mental disorders. It scores each of the nine Diagnostic and Statistical Manual of Mental Disorders, 4th edition, criteria as “0” (not at all) to “3” (nearly every day). High scores for each item represent being frequently bothered by the symptom in the last 2 weeks. The final question assesses overall impairment due to depressive symptoms. A total score of ≥5 indicated that the respondent had mild to severe depression symptoms, while a total score of ≥10 indicated that the respondent had clinical depression or moderate to severe depression symptoms. Depression was defined as a PHQ-9 score ≥ 10 in our analysis [20, 21].

### Sociodemographic characteristics

Sociodemographic characteristics included sex (grouped as male or female), age (< 30, 30–54, 55–74, or ≥ 75 years old), body mass index ([BMI] < 25, 25–30, or ≥ 30 m/kg^2^), race/ethnicity (Mexican American, other Hispanic, Non-Hispanic White, Non-Hispanic Black, or other), marital status (married or unmarried), highest educational level (high school or less, some college, or college graduate or above), taking antidepressants (yes or no), health insurance (yes or no), health insurance cover prescriptions (yes or no) and family income (< 130, 130–349%, or ≥ 350% of the federal poverty level [FPL]) [22]. Family income was categorized using FPL information, which accounts for inflation and family size. The cut point for participation in the Supplemental Nutrition Assistance Program is 130% of the poverty level, and 350% provides equal sample sizes for each of the three income groups. After resting quietly in a sitting position for 5 minutes and determining the maximum inflation level, three consecutive blood pressure readings are obtained. Mean systolic and diastolic blood pressure was obtained using the NHANES recommended method [23]. All were collected as part of the NHANES home questionnaire or physical examination in the mobile examination center, using standardized techniques and equipment.

### Medication adherence

**Fig. 1:**
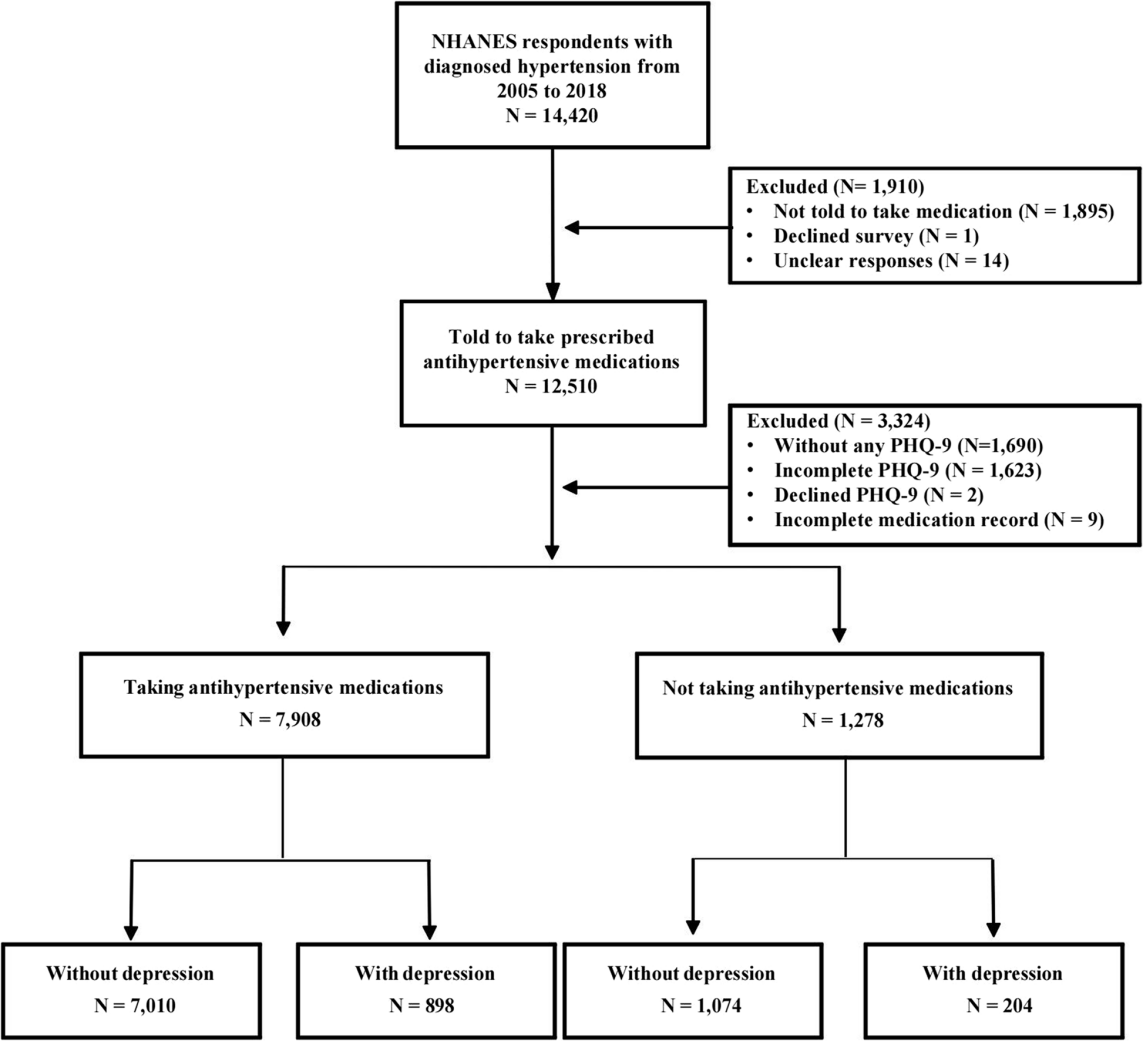
Screening flow of respondents included in research. Abbreviation: NHANES, National Health and Nutritional Examination Survey; PHQ, Patient Health Questionnaire

Data from the blood pressure & cholesterol section and prescription medications section of the NHANES questionnaire were used to assess medication adherence [24]. According to the blood pressure interview, we included patients with hypertension whom professionals advised to take antihypertensive medications (Fig. 1). During the prescription medications section, respondents are asked if they have taken medications in the past 30 days for which they needed a prescription. Those who answer “yes” are asked to show the interviewer the medication containers of all the products used. For each medication reported, the interviewer enters the product’s complete name from the container into a computer. The respondents will be considered to be taking prescribed antihypertensive medications if these medications contain antihypertensive medications. We defined good medication adherence as taking prescribed antihypertensive medications that the respondent has been advised to abide by a doctor. Poor medication adherence was defined as being told of the need to take prescribed antihypertensive medications but not currently taking them [4, 16].

### Statistical methods

Statistical analysis was carried out using SPSS version 26. Quantitative variables were described using mean and standard deviation. Qualitative variables were defined using frequency with percentage. Quantitative variables were tested using the Shapiro-Wilk test to determine the type of distribution. Comparisons between groups were conducted using the Student’s t-test, the Mann-Whitney U test for quantitative characteristics, and the chi-square test or the Fisher exact test for qualitative characteristics. In the univariate analysis, multiple testing corrections adjust *p*-values derived from multiple statistical tests to correct for the occurrence of false positives, and the significance level was set at Bonferroni corrected *p* < 0.05/14 (the number of comparisons) = 0.0035. Due to the large number of risk factors investigated in this study, only elements with p < 0.05 in the univariable analysis were included in the multivariable logistic regression analysis. Model 1 was a multivariate logistic regression model including clinical and socioeconomic factors. Model 2 had demographic characteristics in multivariable logistic regression analysis. Model 3 was a multivariate logistic regression model including all mentioned control variables. A *p*-value of < 0.05 was considered statistically significant.

## Results

Twelve thousand five hundred ten NHANES respondents (2005–2018) had been told to take prescribed antihypertensive medications. After excluding data from 3324 respondents with incomplete medication records or incomplete PHQ-9 questionnaires, data for 9186 were eventually included in the analysis. Of these, 7908 (86.1%) respondents had good medication adherence (898 with depression and 7010 without), and 1278 (13.9%) had poor medication adherence (204 with depression and 1074 without; Fig. 1). The missing data of the included respondents are shown in eTable 1.

### Respondent characteristics

A summary of respondent characteristics for those who were and were not adherent to prescribed antihypertensive medication is shown in Table 1. When the groups were compared, significant differences in adherence were seen by sex, age, BMI, race/ethnicity, marital status, family income, systolic blood pressure (SBP), diastolic blood pressure (DBP), taking antidepressants, health insurance, depression score, and status. Numerically higher proportions of good medication adherence were female, ≥55 years of age, with a BMI ≥25, non-Hispanic White ethnicity, were married, and a family income ≥130% of FPL, with health insurance than comparator groups. The group with good medication adherence has lower systolic and diastolic blood pressure. Depression scores were significantly lower in patients with good medication adherence, and the proportion of respondents in each depression severity category was also lower.

**Table 1:** Respondent characteristics by adherence to prescribed antihypertensive medication

	Taking prescribed antihypertensive medication ***N*** = 7908	Not taking prescribed antihypertensive medication ***N*** = 1278	***P***-value
Gender, n respondents (%)	N = 7908	N = 1278	**.002**
Male	3663 (46.3)	651 (50.9)	
Female	4245 (53.7)	627 (49.1)	
Age, n respondents (%)	N = 7908	N = 1278	**<.001**
< 30 years	58 (0.7)	87 (6.8)	
30–54 years	1769 (22.4)	631 (49.4)	
55–74 years	4254 (53.8)	469 (36.7)	
> 75 years	1827 (23.1)	91 (7.1)	
BMI, kg/m^2^, n respondents (%)	*N* = 7774	*N* = 1258	**.006**
Normal: BMI of < 25	1281 (16.2)	252 (19.7)	
Overweight: BMI of 25 to < 30	2507 (31.7)	376 (29.4)	
Obese: BMI ≥30	3986 (50.4)	630 (49.3)	
Race/ethnicity, n respondents (%)	N = 7908	N = 1278	**<.001**
Mexican American	775 (9.8)	191 (14.9)	
Other Hispanic	566 (7.2)	114 (8.9)	
Non-Hispanic White	3742 (47.3)	498 (39.0)	
Non-Hispanic Black	2236 (28.3)	371 (29.0)	
Other	589 (7.4)	104 (8.1)	
Married, n respondents (%)	4360 (55.1)	575 (45.0)	**<.001**
Educational level, n respondents (%)	*N* = 7896	*N* = 1266	.036
High school or less	4131 (52.2)	687 (53.8)	
Some college	2265 (28.6)	377 (29.5)	
College Graduate or above	1500 (19.0)	202 (15.8)	
Family income, n respondents (%)	*N* = 7249	*N* = 1155	**<.001**
< 130% of FPL	2102 (26.6)	469 (36.7)	
130–349% of FPL	2947 (37.3)	406 (31.8)	
≥ 350% of FPL	2200 (27.8)	280 (21.9)	
SBP, mmHg, median (quartile)	131 (119,144)	133 (120,148)	**<.001**
DBP, mmHg, median (quartile)	71 (62,79)	77 (68,86)	**<.001**
Health insurance	*N* = 7901	N = 1278	**<.001**
Yes	7252 (91.8)	897 (70.2)	
No	649 (8.2)	381 (29.8)	
Health insurance cover prescriptions	*N* = 7264	*N* = 890	.136
Yes	6676 (91.9)	805 (90.4)	
No	588 (8.1)	85 (9.6)	
Taking antidepressants	*N* = 7880	*N* = 1273	**<.001**
Yes	1358 (17.2)	105 (8.2)	
No	3522 (82.8)	1168 (91.8)	
PHQ-9 score, median (quartile)	2.00 (0.00,5.00)	3.00 (0.00,7.00)	**<.001**
Depression status, n respondents (%)	N = 7908	N = 1278	**<.001**
Without depression	7010 (88.6)	1074 (84.0)	
Depression	898 (11.4)	204 (16.0)	

*BMI* body mass index; *FPL* federal poverty level; *PHQ* Patient Health Questionnaire; *SD* standard deviation; *SBP* systolic blood pressure; *DBP* diastolic blood pressure

Significant differences are shown in bold

### The depression symptoms and medication adherence

**Fig. 2:**
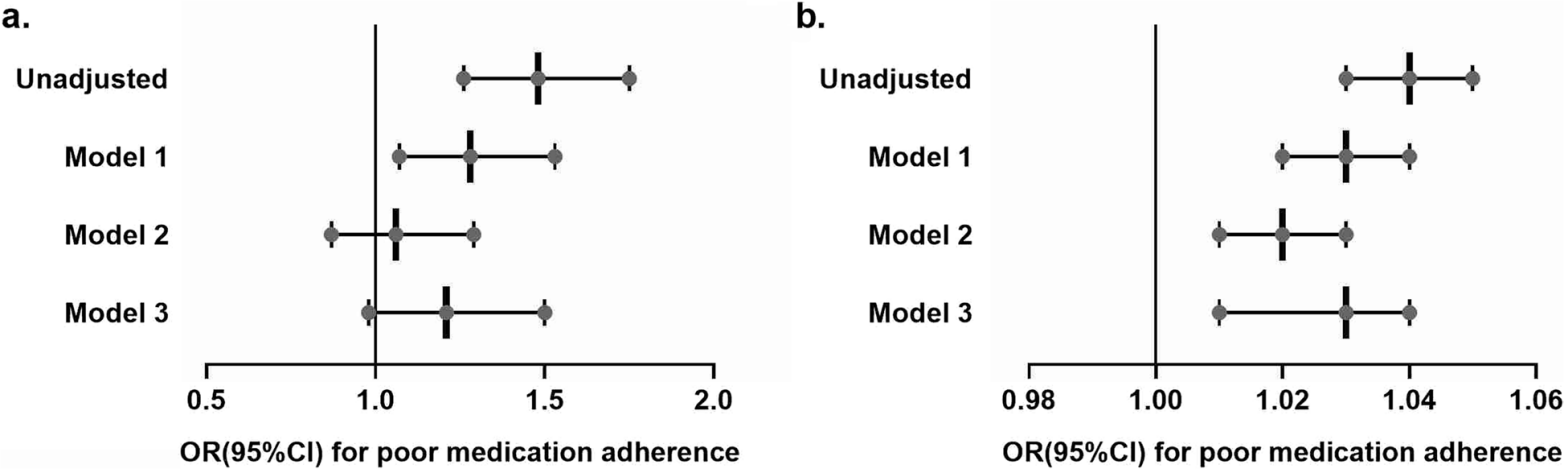
Associations of depression with poor medication adherence. **a** Associations of the depression status with poor medication adherence; **b** Associations of the PHQ-9 scores with poor medication adherence; ^*^ Model 1 adjusted for SBP, DBP, family income, and health insurance; ^*^ Model 2 adjusted for sex, age, educational level, marital status, BMI, and race; ^*^ Model 3 adjusted for sex, age, educational level, marital status, BMI, race, family income, SBP, DBP, and health insurance; Abbreviation: OR: Odds ratio; CI: confidence interval; PHQ, Patient Health Questionnaire; BMI, body mass index; SBP, systolic blood pressure; DBP, diastolic blood pressure

The association between depression and medication adherence was presented in Table 2 and Fig. 2. Poor medication adherence was associated with depression (odds ratio [OR]: 1.48, 95% confidence interval [CI]: 1.26 to1.75, *p* < 0.001) in the unadjusted analyses. Depression was positively associated with poor medication adherence after adjusted clinical and socioeconomic factors (adjusted odds ratio [aOR]: 1.28, 1.07 to1.53, *p* = 0.006, model 1), including systolic blood pressure, diastolic blood pressure, family income, and health insurance. But, there was no significant statistical correlation between depression and poor medication adherence after adjusted demographic factors (aOR: 1.06, 0.87 to 1.29, *p* = 0.55, model 2; aOR: 1.21, 0.98 to 1.50, *p* = 0.075, model 3), included gender, age, educational level, marital status, BMI, race, family income, SBP, DBP, taking antidepressants and health insurance. In unadjusted analyses, we found a significant correlation between PHQ-9 score and poor medication adherence (OR: 1.04 per each point increase, 95% CI; 1.03 to 1.05, *p* < 0.001), with respondents with more severe depression having poorer adherence. When analyses were adjusted for sex, age, educational level, marital status, BMI, race, family income, SBP, DBP, taking antidepressants, and health insurance, the correlation between depression score and adherence was still statistically significant (aOR: 1.03 per each point increase, 1.01 to 1.04, *p* < .001, model 3). To figure out the associations between depression and other characteristics and significance in clinical outcomes, we further compared the baseline characteristics between groups categorized by depression (eTable 2). The results indicated that depression was affected by sex, age, educational level, marital status, BMI, race, family income, and health insurance (*p* < .001).

**Table 2:** Association between depression and antihypertensive medication adherence

	Risk of poor medication adherence							
	Unadjusted		Model 1^a^		Model 2^b^		Model 3^c^	
	OR (95%CI)	P for value	OR (95%CI)	P for value	OR (95%CI)	P for value	OR (95%CI)	P for value
Depression status								
Without depression	Ref.		Ref.		Ref.		Ref.	
Depression	**1.48 (1.26,1.75)**	**<.001**	**1.28 (1.07,1.53)**	**.006**	1.06 (0.87,1.29)	.545	1.21 (0.98,1.50)	.075
PHQ-9 score (per each point increase)	**1.04 (1.03, 1.05)**	**<.001**	**1.03 (1.02,1.04)**	**<.001**	**1.02 (1.01,1.03)**	**.023**	**1.03 (1.01,1.04)**	**<.001**

*CI* confidence interval; *OR* odds ratio; *SBP* systolic blood pressure; *DBP* diastolic blood pressure

^a^Adjusted for SBP, DBP, family income, and health insurance

^b^Adjusted for gender, age, educational level, marital status, BMI, and race

^c^Adjusted for gender, age, educational level, marital status, BMI, race, family income, SBP, DBP, taking antidepressants, and health insurance

Significant differences are shown in bold

### The sociodemographic characteristics and medication adherence

We examined the correlation between sociodemographic characteristics and antihypertensive medication adherence (Table 3). In unadjusted analyses, being female (vs. male), having a higher BMI, older age, a non-Hispanic White or Black race/ethnicity (vs. Mexican American), being married (vs. unmarried), having a college graduate or above (vs. high school or less), having a higher family income, taking antidepressants (vs. not taking antidepressants) and having health insurance (vs. without health insurance) were associated with good medication adherence (*p* < 0.05). Except for the highest educational level and family income, these factors were also significant in the adjusted analyses, with similar OR.

**Table 3:** Risk of antihypertension non-adherence by sociodemographic characteristics

	Risk of poor medication adherence			
	Crude OR (95% CI)	*P* value	Adjusted OR^a^ (95% CI)	*P* value
Gender				
Male	Ref.		Ref.	
Female	**0.83 (0.74,0.94)**	**.002**	**0.77 (0.67,0.89)**	**<.001**
Age^b^ (per one year old increase)	**0.94 (0.93,0.94)**	**<.001**	**0.94 (0.93,0.94)**	**<.001**
BMI, kg/m^2^				
BMI of < 25	Ref.		Ref.	
BMI of 25 to < 30	**0.76 (0.64,0.91)**	**.002**	**0.67 (0.55,0.83)**	**<.001**
BMI ≥30	**0.80 (0.69,0.94)**	**.007**	**0.54 (0.45,0.66)**	**<.001**
Race/ethnicity				
Mexican American	Ref.		Ref.	
Other Hispanic	0.82 (0.63,1.06)	.122	1.03 (0.76,1.34)	.844
Non-Hispanic White	**0.54 (0.45,0.65)**	**<.001**	**0.71 (0.57,0.89)**	**.003**
Non-Hispanic Black	**0.67 (0.56,0.82)**	**<.001**	**0.68 (0.54,0.86)**	**.001**
Other	**0.72 (0.55,0.93)**	**.013**	0.79 (0.58,1.07)	0.13
Marital status				
Not married	Ref.		Ref.	
Married	**0.67 (0.60,0.76)**	**<.001**	**0.73 (0.63,0.85)**	**<.001**
Educational level				
High school or less	Ref.		Ref.	
Some college	1.00 (0.87,1.15)	0.99	0.93 (0.79,1.09)	.383
College Graduate or above	**0.81 (0.69,0.96)**	**.014**	0.89 (0.72,1.09)	.253
Family Income		**<.001**		.191
< 130% of FPL	Ref.		Ref.	
130–349% of FPL	**0.62 (0.54,0.71)**	**<.001**	0.87 (0.74,1.02)	.085
≥ 350% of FPL	**0.57 (0.49,0.67)**	**<.001**	0.87 (0.71,1.05)	.176
Health insurance				
No	Ref.		Ref.	
Yes	**0.21 (0.18,0.24)**	**<.001**	**0.39 (0.32,0.46)**	**<.001**
Taking antidepressants				
No	Ref.		Ref.	
Yes	**0.43 (0.35,0.53)**	**<.001**	**0.46 (0.36,0.59)**	**<.001**

*OR* odds ratio; *CI* confidence interval; *BMI* body mass index

^a^Adjusted for sex, age, educational level, marital status, race, BMI, family income, taking antidepressants, and health insurance

^b^Age was included as a continuous variable in the logistic regression model

Significant differences are shown in bold

## Discussion

This study found that among US NHANES respondents (2005–2018) with prescribed antihypertensive medication, adherence was marginally worse in those with depression and also influenced by a range of sociodemographic characteristics. These respondents had 1.03 times the odds of being non-adherent per each PHQ-9 point increase, and poor antihypertensive medication adherence was more common in patients with worse depression.

Depression is common in patients with hypertension and is associated with a poorer diet, poorer prognosis, higher functional impairment, and higher healthcare costs [11, 25]. Previous studies have found depression to reduce medication adherence [26]. Specific to patients with hypertension, a cross-sectional study found a significant association between depressive symptoms and poor medication adherence [5]. Further, a cohort study of 2180 elderly patients with hypertension found those with depression and a low level of social support to have significantly worse medication adherence after 1 year [27]. Similar to these findings, our study observed poorer antihypertension medication adherence in patients with depression. Besides, we found that the risk of poor medication adherence is higher in those with higher depression scores. It denotes that the risk of non-adherence is higher in those with more severe depression. We should pay more attention to those hypertension patients with high depression scores and urge them to stick to medication in clinical work. We also demonstrated that the covariates (sex, age, highest educational level, marital status, BMI, race, family income, and health insurance) are associated with depression, which partly explains why the correlation between depression and medication adherence declined after adjustment for these factors. In previous studies, it had been confirmed that sex, age, education level, marital status, weight, race, economic status, and medical insurance are significantly correlated with depression [28–31].

Recent evidence suggests that treating depression might improve medication adherence; however, the effectiveness of standard interventions in reducing medication non-adherence and early discontinuation has been limited [32]. Similar to previous studies, our study confirms that taking antidepressants was negatively associated with medication adherence (OR: 0.46, 0.36 to 0.59, *p* < 0.001). With the increasing incidence of depression in patients with hypertension, it is necessary to treat the whole person to achieve the best outcomes [11]. Clinicians should consider screening for depression in patients with hypertension and provide interventions where appropriate to help optimize medication adherence and patient prognosis [33]. In a randomized pilot trial that integrated depression treatment into the standard care for hypertension, improved adherence to antidepressant and antihypertensive medications improved depression outcomes, and blood pressure control was observed among primary care patients [34]. This outcome may also apply to other chronic diseases, as enhancing mental health care among those with chronic illnesses managed in primary care has been shown to improve outcomes and reduce healthcare costs [35].

We also found that respondents who were female (vs. male), older, of non-Hispanic White or Black race (vs. Mexican American), married (vs. unmarried), with a higher BMI, and with health insurance were more likely to have better antihypertensive medication adherence. These findings are consistent with previous studies. The Take Control of Your Blood Pressure study showed that marriage was associated with better medication adherence [6]. It has been proved that marital status can affect the occurrence and prognosis of cardiovascular diseases, and social causation theory suggests that individuals benefit from spousal support [36]. Moreover, awareness, treatment, and control of blood pressure are worse among a representative sample of noninstitutionalized US adults aged 18–39 years compared to those ≥40 years. Further, access to healthcare could influence the prescription of antihypertensive medications and the degree of primary care follow-up [37]. This factor would be most relevant in young men (who tend to lack insurance and primary care) and individuals with low Social and economic support (SES) [1]. Our findings have found poorer medication adherence in such individuals, younger men without health insurance.

There are several strengths to our study. Firstly, using the NHANES database allowed access to a large, ethnically diverse, and nationally representative sample of respondents. The NHANES is conducted with a standardized methodology, and the data is high quality. This will make our results more stable and convincing. Secondly, using the PHQ-9 scale allowed a validated assessment of depression. Thirdly, our analyses on depression included adjustment for several sociodemographic factors, which were also found to influence medication adherence significantly.

Our study also had some limitations. The main one is that there was no formal assessment of medication adherence. This was inferred from responses to the NHANES questionnaire. Observational data from questionnaires also have an inherent risk of recall bias. In addition, we have yet to explore adherence to medication regimens due to the lack of medication regimen records designed by a professional doctor.

## Conclusion

Depression was associated with poor medication adherence in hypertensive patients, and the correlation increased with depression degree. Variables confirmed in the logistic regression model as having an independent impact on medication adherence include sex, age, race, marital status, BMI, and health insurance.

## Supplementary Information

**Additional file 1.**

## Data Availability

The datasets generated and analyzed during the current study are available in the NHANES repository, https://www.cdc.gov/nchs/nhanes/.

## References

[1] Zhang Y, Moran AE. Trends in the Prevalence, Awareness, Treatment, and Control of Hypertension Among Young Adults in the United States, 1999 to 2014. Hypertension (Dallas, Tex: 1979). 2017;70(4):736–42. 10.1161/HYPERTENSIONAHA.117.09801 PMC5657525 28847890

[2] Kochanek KD, Murphy SL, Xu J, Arias E. Deaths: final data for 2017. Natl Vital Stat Rep. 2019;68(9). 32501199

[3] Burnier M (2017). Drug adherence in hypertension. Pharmacol Res.

[4] Burnier M, Egan BM (2019). Adherence in hypertension. Circ Res.

[5] Wang PS, Bohn RL, Knight E, Glynn RJ, Mogun H, Avorn J (2002). Noncompliance with antihypertensive medications: the impact of depressive symptoms and psychosocial factors. J Gen Intern Med.

[6] Trivedi RB, Ayotte B, Edelman D, Bosworth HB (2008). The association of emotional well-being and marital status with treatment adherence among patients with hypertension. J Behav Med.

[7] Vawter L, Tong X, Gemilyan M, Yoon PW (2008). Barriers to antihypertensive medication adherence among adults--United States, 2005. J Clin Hypertens (Greenwich).

[8] DiMatteo MR, Lepper HS, Croghan TW (2000). Depression is a risk factor for noncompliance with medical treatment: meta-analysis of the effects of anxiety and depression on patient adherence. Arch Intern Med.

[9] Kuo P-L, Pu C (2011). The contribution of depression to mortality among elderly with self-reported hypertension: analysis using a national representative longitudinal survey. J Hypertens.

[10] Chowdhury EK, Berk M, Nelson MR, Wing LMH, Reid CM (2019). Association of depression with mortality in an elderly treated hypertensive population. Int Psychogeriatr.

[11] Ademola AD, Boima V, Odusola AO, Agyekum F, Nwafor CE, Salako BL (2019). Prevalence and determinants of depression among patients with hypertension: a cross-sectional comparison study in Ghana and Nigeria. Niger J Clin Pract.

[12] Sandström YK, Ljunggren G, Wändell P, Wahlström L, Carlsson AC. Psychiatric comorbidities in patients with hypertension--a study of registered diagnoses 2009–2013 in the total population in Stockholm County, Sweden. J Hypertens. 2016;34(3). 10.1097/HJH.0000000000000824 26766563

[13] Gabriel A, Zare H, Jones W, Yang M, Ibe CA, Cao Y (2021). Evaluating depressive symptoms among low-socioeconomic-status African American women aged 40 to 75 years with uncontrolled hypertension: a secondary analysis of a randomized clinical trial. JAMA Psychiatry.

[14] Kim MT, Han H-R, Hill MN, Rose L, Roary M (2003). Depression, substance use, adherence behaviors, and blood pressure in urban hypertensive black men. Annals of behavioral medicine: a publication of the Society of Behavioral Medicine.

[15] Schoenthaler A, Ogedegbe G, Allegrante JP (2009). Self-efficacy mediates the relationship between depressive symptoms and medication adherence among hypertensive African Americans. Health Educ Behav.

[16] Berntson J, Stewart KR, Vrany E, Khambaty T, Stewart JC (2015). Depressive symptoms and self-reported adherence to medical recommendations to prevent cardiovascular disease: NHANES 2005-2010. Soc Sci Med.

[17] Maguire LK, Hughes CM, McElnay JC (2008). Exploring the impact of depressive symptoms and medication beliefs on medication adherence in hypertension--a primary care study. Patient Educ Couns.

[18] Hamieh N, Kab S, Zins M, Blacher J, Meneton P, Empana J-P (2021). Depressive symptoms and non-adherence to treatable cardiovascular risk factors' medications in the CONSTANCES cohort. Eur Heart J Cardiovasc Pharmacother.

[19] prevention CfDCa. National Health and Nutrition Examination Survey 2022 [updated March 4, 2022. Available from: https://www.cdc.gov/nchs/nhanes/index.htm.

[20] Kroenke K, Spitzer RL, Williams JB (2001). The PHQ-9: validity of a brief depression severity measure. J Gen Intern Med.

[21] Levis B, Benedetti A, Thombs BD (2019). Accuracy of patient health Questionnaire-9 (PHQ-9) for screening to detect major depression: individual participant data meta-analysis. BMJ (Clinical research ed).

[22] Ogden CL, Fakhouri TH, Carroll MD, Hales CM, Fryar CD, Li X (2017). Prevalence of obesity among adults, by household income and education - United States, 2011-2014. MMWR Morb Mortal Wkly Rep.

[23] Ostchega Y, Prineas RJ, Paulose-Ram R, Grim CM, Willard G, Collins D (2003). National Health and nutrition examination survey 1999-2000: effect of observer training and protocol standardization on reducing blood pressure measurement error. J Clin Epidemiol.

[24] Statistics NCfH. NHANES Questionnaire Data 2022 [updated 2022. Available from: https://wwwn.cdc.gov/nchs/nhanes/search/datapage.aspx? Component=Questionnaire.

[25] Ciechanowski PS, Katon WJ, Russo JE (2000). Depression and diabetes: impact of depressive symptoms on adherence, function, and costs. Arch Intern Med.

[26] Hamam MS, Kunjummen E, Hussain MS, Nasereldin M, Bennett S, Miller J (2020). Anxiety, depression, and pain: considerations in the treatment of patients with uncontrolled hypertension. Curr Hypertens Rep.

[27] Krousel-Wood M, Islam T, Muntner P, Holt E, Joyce C, Morisky DE (2010). Association of depression with antihypertensive medication adherence in older adults: cross-sectional and longitudinal findings from CoSMO. Ann Behav Med.

[28] Salk RH, Hyde JS, Abramson LY (2017). Gender differences in depression in representative national samples: Meta-analyses of diagnoses and symptoms. Psychol Bull.

[29] Di Florio A, Putnam K, Altemus M, Apter G, Bergink V, Bilszta J (2017). The impact of education, country, race and ethnicity on the self-report of postpartum depression using the Edinburgh postnatal depression scale. Psychol Med.

[30] Jung SJ, Woo H-T, Cho S, Park K, Jeong S, Lee YJ (2017). Association between body size, weight change and depression: systematic review and meta-analysis. Br J Psychiatry.

[31] Perneger TV, Allaz AF, Etter JF, Rougemont A (1995). Mental health and choice between managed care and indemnity health insurance. Am J Psychiatry.

[32] Simon ST, Kini V, Levy AE, Ho PM (2021). Medication adherence in cardiovascular medicine. BMJ (Clinical research ed)..

[33] Viswanathan M, Golin CE, Jones CD, Ashok M, Blalock SJ, Wines RCM (2012). Interventions to improve adherence to self-administered medications for chronic diseases in the United States: a systematic review. Ann Intern Med.

[34] Bogner HR, de Vries HF (2008). Integration of depression and hypertension treatment: a pilot, randomized controlled trial. Ann Fam Med.

[35] Wan J, Chua EYC, Soon WSW, Xie Y, Tang WE (2021). The impact of a mental health service on chronic disease management in primary care. Singap Med J.

[36] Wong CW, Kwok CS, Narain A, Gulati M, Mihalidou AS, Wu P (2018). Marital status and risk of cardiovascular diseases: a systematic review and meta-analysis. Heart.

[37] Kesselheim AS, Huybrechts KF, Choudhry NK, Fulchino LA, Isaman DL, Kowal MK (2015). Prescription drug insurance coverage and patient health outcomes: a systematic review. Am J Public Health.

