# Supplementary material for: Adherence to prescribed antihypertensive medication among patients with depression in the United States
Source: BMC Psychiatry. 2022 Dec 5;22:764. doi: 10.1186/s12888-022-04424-x (PMC9720997; doi:10.1186/s12888-022-04424-x)
Supplement: Supplementary file 1 — Additional file 1. [file 12888_2022_4424_MOESM1_ESM.docx]

**eTable 1. Missing data of the included respondents**

| **Characteristic** | **Missing values** | |
| --- | --- | --- |
|  | **Taking prescribed antihypertensive medication** | **Not taking prescribed antihypertensive medication** |
| Body mass index | 134 (1.7%) | 20 (1.6%) |
| Marital status | 7 (0.1%) | 9 (0.7%) |
| Educational level | 12 (0.2%) | 12 (0.9%) |
| Family Income | 659 (8.3%) | 123 (9.6%) |
| Smoking status | 77 (1.0%) | 10 (0.8%) |
| SBP | 204 (2.6%) | 28 (2.2%) |
| DBP | 204 (2.6%) | 28 (2.2%) |

**eTable 2. Respondent characteristics by depression.**

|  | **Without depression N = 8,084** | **Depression* N = 1,102** | **P value** |
| --- | --- | --- | --- |
| Sex, n respondents (%) | N=8,084 | N=1,102 | **<.001** |
| Male | 3,923 (48.5) | 391 (35.5) |  |
| Female | 4,161 (51.5) | 711 (64.5) |  |
| Age, n respondents (%) | N=8,084 | N=1,102 | **<.001** |
| <30 years | 117 (1.4) | 28 (2.5) |  |
| 30-54 years | 1,997 (24.7) | 403 (36.6) |  |
| 55-74 years | 4,179 (51.7) | 544 (49.4) |  |
| >75 years | 1,791 (22.2) | 127 (11.5) |  |
| BMI, kg/m^2^, n respondents (%) | N=7,953 | N=1,079 | **<.001** |
| Normal: BMI of <25 | 1,384 (17.1) | 149 (13.5) |  |
| Overweight: BMI of 25 to <30 | 2,623 (32.4) | 260 (23.6) |  |
| Obese: BMI ≥30 | 3,946 (48.8) | 670 (60.8) |  |
| Race/ethnicity, n respondents (%) | N=8,084 | N=1,102 | **<.001** |
| Mexican American | 829 (10.3) | 137 (12.4) |  |
| Other Hispanic | 561 (6.9) | 119 (10.8) |  |
| Non-Hispanic White | 3,777 (46.7) | 463 (42.0) |  |
| Non-Hispanic Black | 2,296 (28.4) | 311 (28.2) |  |
| Other | 621 (7.7) | 72 (6.5) |  |
| Married, n respondents (%) | 4,525 (56.0) | 410 (37.3) | **<.001** |
| Educational level, n respondents (%) | N=8,062 | N=1,100 | **<.001** |
| High school or less | 4,116 (50.9) | 702 (63.7) |  |
| Some college | 2329 (28.8) | 313 (28.4) |  |
| College Graduate or above | 1,617 (20.0) | 85 (7.7) |  |
| Family income, n respondents (%) | N=7,422 | N=982 | **<.001** |
| <130% of FPL | 2,039 (25.2) | 532 (48.3) |  |
| 130%-349% of FPL | 3,019 (37.3) | 334 (30.3) |  |
| ≥350% of FPL | 2,364 (29.2) | 116 (10.5) |  |
| SBP, mmHg, median (quartile) | 131 (120,145) | 129 (117,143) |  |
| DBP, mmHg, median (quartile) | 71 (62,80) | 73 (65,81) |  |
| Health insurance | N=8,078 | N=1,101 | **<.001** |
| Yes | 7,232 (89.5) | 917 (83.2) |  |
| No | 846 (10.5) | 184 (16.7) |  |
| Health insurance cover prescriptions | N=7,232 | N=922 | .158 |
| Yes | 6,624 (81.9) | 857 (77.8) |  |
| No | 608 (7.5) | 65 (5.9) |  |

Abbreviations: BMI, body mass index; FPL: federal poverty level; SBP, systolic blood pressure; DBP, diastolic blood pressure; SD, standard deviation;

^*^Depression was defined as a PHQ-9 score ≥10 in our analysis;

Significant differences are shown in bold.
